# Drought-induced reduction in flower size and abundance correlates with reduced flower visits by bumble bees

**DOI:** 10.1093/aobpla/plab001

**Published:** 2021-01-06

**Authors:** J Kuppler, J Wieland, R R Junker, M Ayasse

**Affiliations:** 1 Institute of Evolutionary Ecology and Conservation Genomics, Ulm University, Ulm, Germany; 2 Evolutionary Ecology of Plants, Department of Biology, Philipps-University Marburg, Marburg, Germany; 3 Department of Biosciences, University of Salzburg, Salzburg, Austria

**Keywords:** Climate change, floral traits, plant–insect interactions, pollination, pollinators, RFID

## Abstract

Reduced water availability can cause physiological stress in plants that affects floral development leading to changes in floral morphology and traits that mediate interactions with pollinators. As pollinators can detect small changes in trait expressions, drought-induced changes in floral traits could affect pollinator visitations. However, the linkage between changes in floral traits and pollinator visitations under drought conditions is not well explored. We, therefore, tested whether drought-induced changes in floral morphology and abundance of flowers are linked to changes in pollinator visitations. We conducted flight cage experiments with a radio frequency identification system for automated visitation recordings with bumble bees (*Bombus terrestris*) and common charlock (*Sinapis arvensis*) as the model system. In total, we recorded interactions for 31 foraging bumble bees and 6569 flower visitations. We found that decreasing soil moisture content correlated with decreasing size of all measured morphological traits except stamen length and nectar tube width. The reductions in floral size, petal width and length, nectar tube depth and number of flowers resulted in decreasing visitation rates by bumble bees. These decreasing visitations under lower soil moisture availability might be explained by lower numbers of flowers and thus a reduced attractiveness and/or by increased difficulties experienced by bumble bees in handling smaller flowers. Whether these effects act additively or synergistically on pollinator behaviour and whether this leads to changes in pollen transfer and to different selectionp ressures require further investigation.

## Teaser

Under dry conditions, plants can reduce their flower number and size to save water. Such changes, however may lead to reduced pollinator visitations as these traits play an important role in pollinator attraction. In this study, we explored how drought affects pollinator visitation via changes in floral traits. Therefore, we conducted a flight cage experiment with an automated visitation recording system observing bumblebee interactions with drought stressed and well-watered plants. Our results show that decreasing flower number and size under drought conditions resulted in decreasing bumblebee visits. This indicates that drought-stressed plants become less attractive for pollinators.

## Introduction

With changing climate, the predicted alterations in precipitation regimes will decrease water availability in many regions across the globe (IPCC 2014). These declines might affect pollinators directly and indirectly (Soroye *et al.* 2020). Direct effects include decreased colonization, declining populations and extinctions in areas of non-suitable climate over time (Sgrò *et al.* 2016; Soroye *et al.* 2020). Indirect effects include declining nesting resources, phenological mismatch (Forrest 2015) or alterations of floral resources and phenotypes (Burkle and Runyon 2016; Waser and Price 2016; Gallagher and Campbell 2017; Phillips *et al.* 2018). As floral traits play a central role in plant–pollinator interactions (Kuppler *et al*. 2016, 2017), their changes can disrupt or modify these interactions with consequences for plants and animals (Burkle and Runyon 2016; Gallagher and Campbell 2017; Glenny *et al.* 2018). Therefore, an understanding of the impact of abiotic conditions on floral traits represents an important puzzle piece in the overall picture of the way that plant–pollinator interactions will be altered by environmental change.

Reduced water availability can cause physiological stress in plants resulting in changes of vegetative and floral phenotypes (Schulze *et al.* 2002; Lambers *et al.* 2008; Teixido *et al.* 2019). Flower production and maintenance is energetically costly in terms of the carbon, nutrients and water supplies needed (Galen 2000; Teixido and Valladares 2014; Roddy *et al*. 2016, 2018, 2019; Gallagher and Campbell 2017). Therefore, plants under reduced water availability often produce smaller, fewer and/or short-lived flowers (Carroll *et al.* 2001; Burkle and Runyon 2016; Teixido *et al.* 2016; Gallagher and Campbell 2017). Whereas these responses have been observed for several plant species, floral traits that are not directly linked to water conservation, such as floral scent, seem to have species-specific responses in terms of magnitude and direction (Burkle and Runyon 2016; Campbell *et al.* 2019). Overall, these phenotypic changes might lead to changes in pollinator visitation and plant–pollinator interactions.

Pollinators are able to detect even small changes in floral traits and adjust their behaviour in order to maximize floral handling and resource usage/intake (Mothershead and Marquis 2000; Dohzono *et al.* 2011). Hence, changes in pollinator visitations can be triggered by intraspecific changes or variation in different floral traits (Conner and Rush 1996; Kuppler *et al.* 2016; Sletvold *et al.* 2016). Lower numbers of flower/inflorescences and, thus, smaller floral displays and fewer resources per plant can reduce visitation rates (Vaughton and Ramsey 1998; Thompson 2001; Mitchell *et al.* 2004; Burkle and Runyon 2016). Smaller floral size can have the same effect (Vaughton and Ramsey 1998; Thompson 2001; Burkle and Runyon 2016) with changes in nectar tube depth and corolla size being especially important. For example, the handling efficiency and resource intake of bumble bees, mediated by tongue length, is often higher on larger and deeper flowers (Harder 1983; Dohzono *et al.* 2011; Klumpers *et al.* 2019). Therefore, we might expect correlations between drought, reduced floral display and size and reduced visitation rates.

This expected correlation between drought and floral display/size has been detected in various studies (Burkle and Runyon 2016; Gallagher and Campbell 2017, but see Glenny *et al.* 2018) but visitation rates seem to vary between showing no effect, peaking at intermediate traits values, increasing with traits values (Burkle and Runyon 2016; Gallagher and Campbell 2017; Glenny *et al.* 2018) or differing between pollinator species (Burkle and Runyon 2016). Therefore, experimental evidence is mixed and direct tests of the linkage between reduced flower visitations and drought-induced changes in floral traits remain scarce.

Flowers are multimodal displays that attract pollinators and ensure pollination (Faegri and van der Pijl 1979; Willmer 2011). Within these displays, groups of floral traits are associated with various functions (Willmer 2011; Junker and Parachnowitsch 2015): pollinator attraction or pollination efficiency, i.e. flower–pollinator fit. Pollinator attraction is usually attributed to floral scent, colour, to floral abundance and partially to morphology (Raguso 2008; Junker and Parachnowitsch 2015), whereas specific morphological traits, such as the position of stamen and stigma or nectar tube width, are directly related to pollen reception or removal and are often critical for successful pollination (Harder and Barrett 1993; Campbell *et al.* 1996; Sletvold and Agren 2011; Opedal 2018). Therefore, specific trait expressions that are necessary to ensure pollination efficiency might remain stable under drought conditions to maintain their function.

With regard to attraction traits, reduced water availability can alter scent composition and emission rates (Burkle and Runyon 2016; Glenny *et al.* 2018; Campbell *et al.* 2019; Rering *et al.* 2020), whereas colour has been shown to remain unaffected in some studies (Arista *et al.* 2013; Brunet and Van Etten 2019). Further, the number of flowers and the size of flowers is often reduced (Elle and Hare 2002; Arista *et al.* 2013; Burkle and Runyon 2016; Glenny *et al.* 2018; Brunet and Van Etten 2019; Walter 2020). The reduction of size includes nectar/corolla tube width (O’Halloran and Carr 2010; Gallagher and Campbell 2017; Mantel and Sweigart 2019, but see Campbell and Wendlandt 2013 and Caruso 2006), although other traits related to pollination efficiency, such as style length or herkogamy, are often less affected by drought or respond inconsistently (Roy and Stanton 1999; Elle and Hare 2002; Halpern *et al.* 2010; Kay and Picklum 2013; Miranda and Vieira 2014; Opedal *et al.* 2016; Mantel and Sweigart 2019).

Here, our aim has been to understand whether changes in floral traits under experimentally simulated fluctuating precipitation are linked to reduced floral visitations and, thus, ultimately affect pollination and potentially plant reproductive success. Our first expectation was that plant individuals under dry conditions are smaller, produce lower numbers of flowers and show decreasing flower size. Our second expectation was that these reductions of traits values, which are important for bumble bee attraction, reduce the number of visits that each plant receives. We tested these predictions by conducting flight cage experiments with a radio frequency identification system (RFID) automatically recording interactions in our model system of bumble bees (*Bombus terrestris*) and common charlock (*Sinapis arvensis*).

## Methods

### Study species and drought treatment

*Sinapis arvensis* L. (Common Charlock, Brassicaceae) is a wide-spread annual self-incompatible plant. It is native to Southern and Middle Europe, occurs mainly on fields, field margin and ruderal areas and is visited by a broad range of flower visitors; mostly hoverflies, bees and bumble bees (Kunin 1993; Hoffmeister *et al.* 2016). Its ecological indicator values for moisture is 3, i.e. relatively moist (range: 1 = very dry to 5 = standing in water), according to Landolt *et al.* (2010) and indifferent according to Ellenberg *et al.* (1992). Its turgor loss point or ‘wilting point’ is −1.74 ± 0.031 MPa following the method of Sun *et al.* (2020) and thus exhibits approximately the mean value of the 41 temperate perennial grassland species (Sun *et al.* 2020). In general, the turgor loss point is a key drought tolerance trait that characterizes the constitutive drought tolerance of most species (Bartlett *et al.* 2014). It is mostly used for woody species but recently has also been applied to herbaceous species (Sun *et al.* 2020).

Our experimental plants were grown in a climate chamber (Phytotron, Vötsch Industrietechnik GmbH, Balingen-Frommern, Germany) in the Botanical Garden of Ulm University, Germany. Seeds from a natural population in Southern Germany (Rieger-Hoffman GmbH, Blaufelden-Raboldshausen, Germany) were placed on filter paper soaked with aqueous gibberelic acid solution (1000 ppm; Roth, Karlsruhe, Germany) and maintained under complete darkness at room temperature until germination. Seedlings were transferred to pots (10 × 10.5 × 10.5 cm) filled with a soil mixture of 3:2:1 TKS2:compost:sand (TKS2, Floragard Vertriebs-GmbH, Oldenburg, Germany). After 1 week in a greenhouse, the pots were moved to a climate chamber (12-h photoperiod with 500 μmol m^−2^, 21 °C, 60 % relative humidity). Each week, 30 plants were placed in the climate chamber in order to obtain a succession of flowering individuals.

At the on-set of floral buds, half of these plants (*N* = 15) were randomly placed under a pulsed water treatment to induce intermittent drought stress (Huberty and Denno 2004), as continuous stress might impede flower development (Burkle and Runyon 2016). Individuals in the control group (= watered treatment) were watered daily with 75 mL water, whereas individuals within the drought treatment were watered every third day with the same amount of water; the start of wilting usually occurred on the third day without water. Each plant was treated for at least 10 days before being used in the flight cage experiment. Soil moisture was controlled using a custom-made soil moisture sensor with the Arduino system (Iduino ME110, Arduino software version 1.8.8, board: Genuino Uno; ElectroPeak 2019) calibrated with a soil moisture sensor (Hydrosense 2, Campbell Instruments, Bremen, Germany).

### Floral traits

We measured 11 floral traits for each plant: plant height [mm], number of flowers and inflorescences, diameter of flowers and inflorescences [mm], petal length and width [mm], nectar tube depth and width [mm], stamen length [mm] and style length [mm] (Stang *et al.* 2006; Junker *et al.* 2013; Kuppler *et al*. 2016, 2017). All measurements were made with a calliper (precision ±0.2 mm), except for plant height for which a folding ruler was used. Each plant was measured on the day that it was used in the flight cage experiment.

### Bumble bees

*Bombus terrestris* was used as it is a common flower visitor of *Sinapis arvensis*. The bees were obtained from a self-reared colony at the Institute of Evolutionary Ecology and Conservation Genomics, Ulm University, Germany. The colony was kept in a wooden box (L 39 × B 16.5 × H 16 cm) in constant darkness at a temperature of 26 °C and a relative humidity of 60 %. At the start of the flight cage experiment, the colony was 2 months old and contained approximately 30 workers (for a detailed description of the rearing of bumble bees, see Rottler *et al.* (2013) and Rottler-Hoermann *et al.* (2016).

### Flight cage experiments

Experiments were conducted in a flight cage (2.9 × 1.8 × 2.8 m) in the Botanical Garden of Ulm University, Germany. The flight cage was connected to a two-room container in which one room was kept in constant darkness and at ~26 °C and 60 % relative humidity. The bumble bee colony was placed in this room and was connected to the flight cage with a darkened plastic tube. After two days of acclimatization, every forager (i.e. bumble bee in the flight cage) was caught and a unique RFID-marker (radio frequency identification system, Bee Transponder, GiS mbH, Lenningen, Germany) was glued onto its back by instant glue (Katzenberger *et al.* 2013). In total, we marked 85 foragers, but only 31 visited flowers during the course of the experiment.

To explore whether watered and drought plants received a different number of visits, we placed two blocks of plants 1.5 m apart in the flight cage on each sampling day (*N* = 9). Each block consisted of four plants placed 34 cm apart with one block containing treated plants and the other control plants in order to provide bumble bees with a clear choice between treatments. Blocks were switched every day to avoid position effects. One inflorescence of each plant was equipped with a copper antenna connected to an RFID-reader system (TSR-64, GiS mbH, Lenningen, Germany). Each antenna recorded every RFID-marker and its’ ID within a radius of 1.2 cm (Katzenberger *et al.* 2013). Plants were recorded for 4 h on each sampling day.

### Statistical analysis

As bumble bees moved around on a given monitored inflorescence, antennae recorded each bumble bee multiple times at the same flower. As these recording cannot be viewed as independent flower visits, we removed all multiple recordings at the same plant directly following the initial recording. Therefore, only switches between plants were counted as one flower visit.

To check our drought treatment, we test whether the measured soil moisture content differed between treatments. Therefore, we fitted a linear mixed model with treatment as the fixed factor and date of measurement as the random factor. To test for broad differences in the number of interactions between treatments, we used a negative binomial-distributed generalized linear mixed model with treatment as the fixed factor and date of measurement and block as the random factor. To explore the association between soil moisture and trait expression, we used a generalized linear model either with Gaussian or negative binomial distribution if traits were counts. As soil moisture showed a high variability within the drought treatment (mean (SD): 23.1 (13.5) %), we treated soil moisture as a continuous variable rather than treatment as a categorical variable (Waser and Price 2016; Gallagher and Campbell 2017; the categorical analysis is shown in Supporting Information 1). The association between the number of interactions, floral trait expression and treatment was explored using negative binomial-distributed generalized linear mixed models with treatment and all floral traits and their interaction with treatment as a fixed factor and date of sampling and plants within block nested in block as random factors. All floral trait measurement were centred and scaled using the *scale*(centre = TRUE, scale = TRUE) function. For model simplification, we removed one explanatory variable step by step, starting with the interaction effect with the highest *P*-value. After each step, we compared both models by using a likelihood ratio test to determine whether model simplification was justified. Stepwise deletion stopped when the removal of an explanatory variable was not justified (Crawley 2009). Models with single traits are shown in Supporting Information 2. All models were fitted in *R* 4.0.2 (R Core Team 2020) by using the g*lmmTMB*() function of the *glmmTMB*-package 1.0.2.1 (Brooks *et al.* 2017) and *glm*()/*glm.nb*() function of the *MASS*-package 7.3–51.6 (Venables and Ripley 2002). Significance was ascertained *via* Type II Wald chi-square tests implement in the *Anova*() function of the car package 3.0–10 (Fox and Weisberg 2019). The fit of each model was assessed using the *DHARMa* package 0.3.2.0 (Hartig 2020) and all assumptions for dispersion, zero-inflation, heteroscedasticity, outliers and deviation from the error distribution were met. To explore the co-variation between trait expressions, we calculated the correlation between traits by using Pearson correlation.

## Results

The mean soil moisture content was higher in the control than in the treatment plants (mean (SD): 40.4 (2.89) %; LMM: *Χ*_1_ = 111.76, *P* < 0.001), although we found considerable variation (mean (SD): 23.1 (13. 5) %) within the drought stress treatment. In total, we recorded 6569 interactions of 31 bumble bees with plants. Per day, we recorded 729.9 (297.2) (mean (SD)) interactions and 92.5 (42.3) interactions per plant. The mean number of interactions per plant tended to be higher for watered than drought stress plants (mean (SD): 96.4 (46.1) (watered); 88.5 (38.3) (drought stress); GLMM: *Χ*_*1*_ = 1.67, *P* = 0.19).

**Figure 1.**
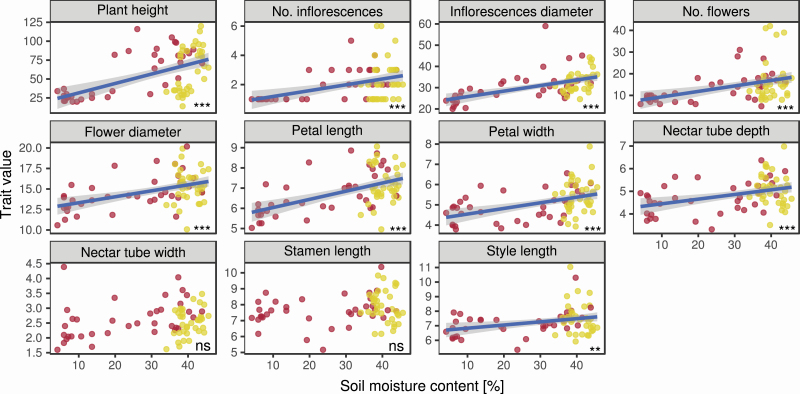
Association between soil moisture content [%] and floral trait expressions of *Sinapis arvensis*. Each red dot represents one plant under drought treatment, each yellow dot one plant under watered treatment. Blue lines are regression lines derived from a (generalized) linear mixed model; grey shades are standard error of the regression lines. No. indicates “Total number of”. Trait values for plant height are in centimetres, No. inflorescences and flowers are counts, other values are in millimetres. Significance levels are given as asterisks: **P < 0.01, ***P < 0.001, ns = not significant. Full model results are given in Supporting Information **Table S1**.

**Figure 2.**
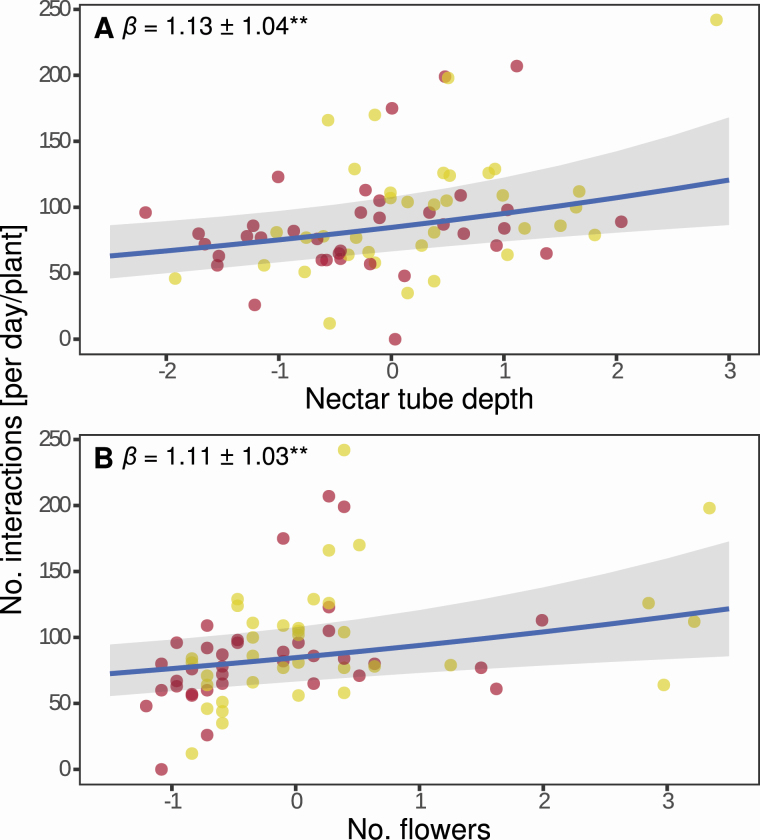
Association between nectar tube depth (A) and number of flowers (B) of *Sinapis arvensis* and total number of interactions per day and plant with *Bombus terrestris*. Each red dot represents one plant under drought treatment, each yellow dot one plant under watered treatment. Blue lines are regression lines derived from (generalized) linear mixed models including trait, treatment and their interaction; grey shades are standard error of the regression lines. No. indicates “Total number of”. Estimate (β) ± standard error are shown. All traits values combined were mean-centred and scaled by one standard deviation. Significance levels are given as asterisks: **P < 0.01. Full model results are given in Supporting Information **Table S2**.

Therefore, we first explored the association of soil moisture content and trait measurement. All traits measured, except for nectar tube width and stamen length, increased with increasing soil moisture content (Fig. 1; **Supporting Information Table S1**). Along our soil moisture gradient (10–40 %) predicted plant height increased by ~55 %, number of inflorescences by ~50 %, inflorescence diameter by ~24 %, number of flowers by ~46 %, flower diameter by ~14 %, petal length by ~17 %, petal width by ~15 %, nectar tube depth by ~12 % and style length by ~9 % (differences in the mean value of the categorical treatment are shown in **Supporting Information File 1**). Further, nectar tube width and stamen length increased by ~11 and 6 %; however, this effect was only marginally significant (see **Supporting Information Table S1**, *P* < 0.1). The increasing trait values of nectar tube depth and number of flowers were associated with an increase in interactions per plant per day (Fig. 2; **Supporting Information Table S2**).

**Figure 3.**
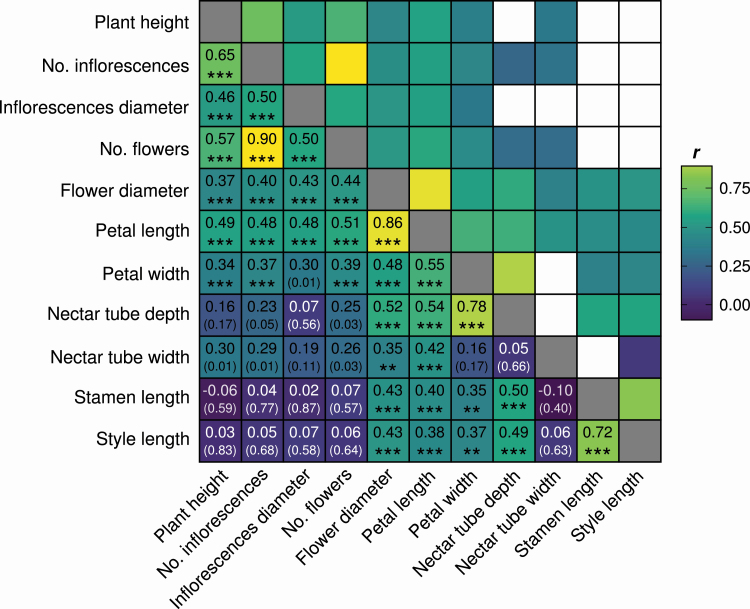
Heatmap of Pearson’s product–moment correlation coefficients *r* between floral traits of *Sinapis arvensis* individuals. In dark squares white text was used to increase visibility. In the upper right half of the matrix, only significant correlations (*P* < 0.05) are shown. In the lower half, all *r*- (large number) and *P-*values (small number in brackets, **P < 0.01 and ***P < 0.001) are shown. No. indicates “Total number of”.

The similar responses of floral traits to soil moisture were also reflected in the correlations between traits (Fig. 3). As excepted, traits that reflected the number of flowers or size of floral display, i.e. plant height, number of inflorescences and flowers and diameter of inflorescences and flowers were correlated. Similarly, traits related to floral size were also correlated with each other. Only stamen and style length showed no correlation with plant height, number of inflorescence and flowers and inflorescence diameter (Fig. 3).

## Discussion

Under reduced water availability, plants decrease water loss *via* phenotypic changes (Schulze *et al.* 2002). These trait changes, however, might also impact other functions of the same trait (Galen 2000; Gallagher and Campbell 2017; Roddy 2019). The floral phenotype is essential for the attraction of pollinators and, thus, drought-induced changes might affect pollinator visitations and pollination. In the experiment reported here, we have shown that reduced soil moisture content results in reduced flower availability, floral display and floral size with lower number of flowers and shallower nectar tubes being linked to a reduced number of visits by bumble bees. Overall, the mean number of visitations was lower in plants under drought conditions while trait variability was higher. The higher variation can be explained by higher variability in soil moisture content in drought compared with watered conditions. Thus, visitation patterns are more closely linked to changing floral trait expression along a soil moisture gradient than to binary response to treatment. Our results reveal that drought-induced phenotypic changes in floral trait expressions also reduce floral visitations, which, in turn, might lead to reduced reproductive output.

The studied plants showed the expected reduction in flower abundance, floral height and several measures of floral size, e.g. petal length and flower diameter, when exposed to declining soil moisture content and when compared with well-watered plants. This is most likely a phenotypic response to reduce water loss under reduced resource availability (Carroll *et al.* 2001; Teixido *et al*. 2016, 2019; Gallagher and Campbell 2017; Glenny *et al.* 2018; Roddy 2019). However, three traits remained largely constant across the soil moisture gradient: increase of nectar tube width and stamen length was not different from zero and style length showed the lowest change per unit of soil moisture. Stamen and style length are relevant for female and male reproductive success and their position is linked to pollination efficiency. Thus, changes in specific expression of these traits might lead to reduced pollination success (Campbell 1989; Young and Stanton 1990; Conner and Rush 1996; Willmer 2011), unless the relative positions of the stamen and style to other floral parts is important for pollination efficiency.

In our study system, the above might occur if the style is too short to contact the pollinator body. Based on their behaviour on flowers, this is more likely for syrphid flies than bumble bees. When syrphid flies forage for pollen their bodies are usually a few millimetres above the floral surface depending on their leg length (pers. obs. J. Kuppler). Thus, in plant species, in which the pollination success depends on a diverse set of pollinators preferring a variety of floral trait expressions, such phenotypic changes should be less important for the pollination success than in more specialized plant species. Nevertheless, under drought, only certain regions of the flower are reduced, whereas others might be kept constant to maintain pollination success.

Our results indicate that the phenotypic expression of floral traits associated with pollinator attraction are more strongly affected by drought than efficiency traits. This has been shown for various efficiency traits and for several specialized and generalized plant species (Roy and Stanton 1999; Elle and Hare 2002; Halpern *et al.* 2010; Opedal *et al.* 2016). Trait expression might also be altered under drought because of changes in mating strategies. For example, selfing rates might increase *via* a reduction in herkogamy (Opedal *et al.* 2016). However, the specific traits that are affected and the magnitude of the effects are most likely dependent on the plant species, population, abiotic conditions and pollinator community.

Traits also showed an expected degree of co-variation. Whereas floral size decreased in concert with the number of flowers, stamen and style length showed no correlation with the number of flowers, but with measures of floral size. This indicates that floral traits are not decoupled from each other (Berg 1960; Armbruster *et al.* 1999), but the precise relationship depends on the traits being considered. Therefore, floral expression seems to be limited to the extent to which traits can vary independently and thus, potentially constrain phenotypic responses to drought, thereby allowing plants to maintain plant–pollinator interactions and efficient pollination. For example, nectar tube width and depth are closely linked to pollinator–flower fit (e.g. Galen 1996; Stang *et al.* 2006; Whittall and Hodges 2007; Klumpers *et al.* 2019) but, if there is a strong selection pressure for a reduction of water loss, the corolla area decreases despite the negative consequences for pollinator–flower fit. This potential conflict needs to be further tested experimentally to understand the potential impact of drought on plants.

In general, plants under drought conditions received a lower number of visitations than control plants indicating an influence of drought on visitation patterns. This is consistent with the findings of previous studies, although different visitor species can respond differently to drought-affected plants (Burkle and Runyon 2016). In our study, the reduced visitation by bumble bees can be explained by smaller flowers and decreasing floral display. Bumble bees have been shown to prefer larger flowers (Inouye 1980; Willmer 2011) due to lower foraging efficiency in smaller flowers because of size mismatch (Klumpers *et al.* 2019). Additionally, plants with higher water availability might produce larger amounts of nectar and therefore be more attractive (Waser and Price 2016), an effect that we cannot distinguish between.

These changes in visitation patterns might have profound consequences for indirect effects of increasing droughts attributable to climate change. First, bumble bees may switch to other plant species to make flower handling more efficient or might not switch and become less efficient. Both may nevertheless decrease the overall foraging efficiency because of the reduced amount of nectar collected and changes in nectar or pollen composition between plant species. Second, if smaller flowers become more prominent, the tongue length of the bumble bees might decrease and/or short-tongued bumble bees might become more dominant as shown in the Rocky Mountains (Miller-Struttmann *et al.* 2015). Thus, bumble bees will be affected if increasing droughts lead to changes in floral trait expression.

In conclusion, our data show that plants under reduced water availability are smaller, produce lower number of flowers and show decreasing floral size. These patterns are more a function of the soil moisture gradient than a binary response to treatment. As expected, the reduction of trait values is linked to lower visitation by bumble bees. Therefore, indirect effects of drought on pollinators can modify plant–pollinator interactions and potentially change pollen transfer and, thus, pollination in plant communities.

## Supporting Information

The following additional information is available in the online version of this article—

**Table S1.** Results of generalized linear models (GLM) testing for differences in floral trait expression of *Sinapis arvensis* across a soil moisture gradient

**Table S2.** Results of the final generalized linear mixed-models (GLMMs) testing for differences in number of interactions of *Bombus terrestris* with *Sinapis arvensis*

**Supporting Information 1.** Binary analysis of floral traits expression between drought and watered treatment

**Supporting Information 2.** Association of interaction with floral expressions for each trait separately

plab001_suppl_Supplementary_Materials

## Data Availability

Data and R-Code used in this paper are available on Figshare https://doi.org/10.6084/m9.figshare.13436432

## Notes

**Form & Function Chief Editor:** Kate McCulloh
