# Supplementary material for: Drought-induced reduction in flower size and abundance correlates with reduced flower visits by bumble bees
Source: AoB Plants. 2021 Jan 6;13(1):plab001. doi: 10.1093/aobpla/plab001 (PMC7891244; doi:10.1093/aobpla/plab001)
Supplement: plab001_suppl_Supplementary_Materials [file plab001_suppl_supplementary_materials.pdf]

Supporting Information

Kuppler J, Wieland J, Junker RR & Ayasse M (2020) **Drought-induced reduction in flower size and abundance correlates with reduced flower visits by bumble bees**. *AoBPlants*

Table S1. Results of generalized linear models (GLM) testing for differences in floral trait expression of *Sinapis arvensis* across a soil moisture gradient. Estimate shows coefficient and 95% Confidence Intervals; values for models with negative binomial error are back-transformed. Significance was assessed using Analysis of Deviance Table (Type II).

Trait	Estimate	*t*	*P*	X_1	*P*
Intercept	19.17 (3.58-34.77)	2.41	0.02		
Plant height	1.25 (0.79-1.70)	5.38	< 0.001	28.90	< 0.001
Intercept	0.79 (0.10-1.48)	2.23	0.03		
Number of inflorescences	0.04 (0.02-0.06)	3.87	< 0.001	14.95	< 0.001
Intercept	23.17 (19.83-26.52)	13.55	< 0.001		
Diameter of inflorescences	0.26 (0.16-0.36)	5.23	< 0.001	27.37	< 0.001
Intercept	7.58 (5.65-10.17)	13.62	< 0.001		
Number of flowers	1.02 (1.01-1.03)	4.772	< 0.001	22.24	< 0.001
Intercept	12.59 (11.44-13.74)	21.46	< 0.001		
Diameter of flowers	0.07 (0.04-0.11)	4.26	< 0.001	18.13	< 0.001
Intercept	5.63 (5.11-6.16)	21.08	< 0.001		
Petal length	0.04 (0.03 - 0.06)	5.20	< 0.001	27.03	< 0.001
Intercept	4.27 (3.82-4.71)	18.74	< 0.001		
Petal width	0.03 (0.01-0.04)	4.20	< 0.001	17.57	< 0.001
Intercept	4.24 (3.83-4.65)	20.11	< 0.001		
Nectar tube depth	0.02 (0.009-0.032)	3.36	0.001	11.32	< 0.001
Intercept	2.28 (1.95-2.61)	13.629	< 0.001		
Nectar tube width	0.009 (-0.0004-0.018)	1.87	0.07	3.51	0.06
Intercept	7.25 (6.67-7.82)	24.75	< 0.001		
Stamen length	0.015 (-0.0014-0.032)	1.80	0.08	3.23	0.08
Intercept	6.61 (3.58-34.78)	23.29	< 0.001		
Style length	0.02 (0.79-1.70)	2.65	0.01	7.00	< 0.01

Table S2. Results of the final generalized linear mixed-models (GLMMs) testing for differences in number of interactions of *Bombus terrestris* with *Sinapis arvensis*. Estimate shows coefficient and 95% Confidence Intervals; values are back-transformed

**Trait**	**Estimate**	***z***	***P***	$\boldsymbol{X_1}$	***P***
Intercept	84.71 (66.6-107.7)	36.19	< 0.001		
Nectar tube depth	1.13 (1.04-1.21)	2.95	0.0032	*8.70*	0.0032
Number of flowers	1.11 (1.03-1.19)	2.75	0.0059	7.57	0.0059

The full model included all traits, treatment and the interaction between trait and treatment as fixed factors and plants within block nested in block and sampling date as random factors. Final model after stepwise model simplification are shown. ***, $P < 0.001$; **, $P < 0.01$; significance of fixed effects was assessed with likelihood ratio tests.

Supporting Information 1. Binary analysis of floral traits expression between drought and watered treatment

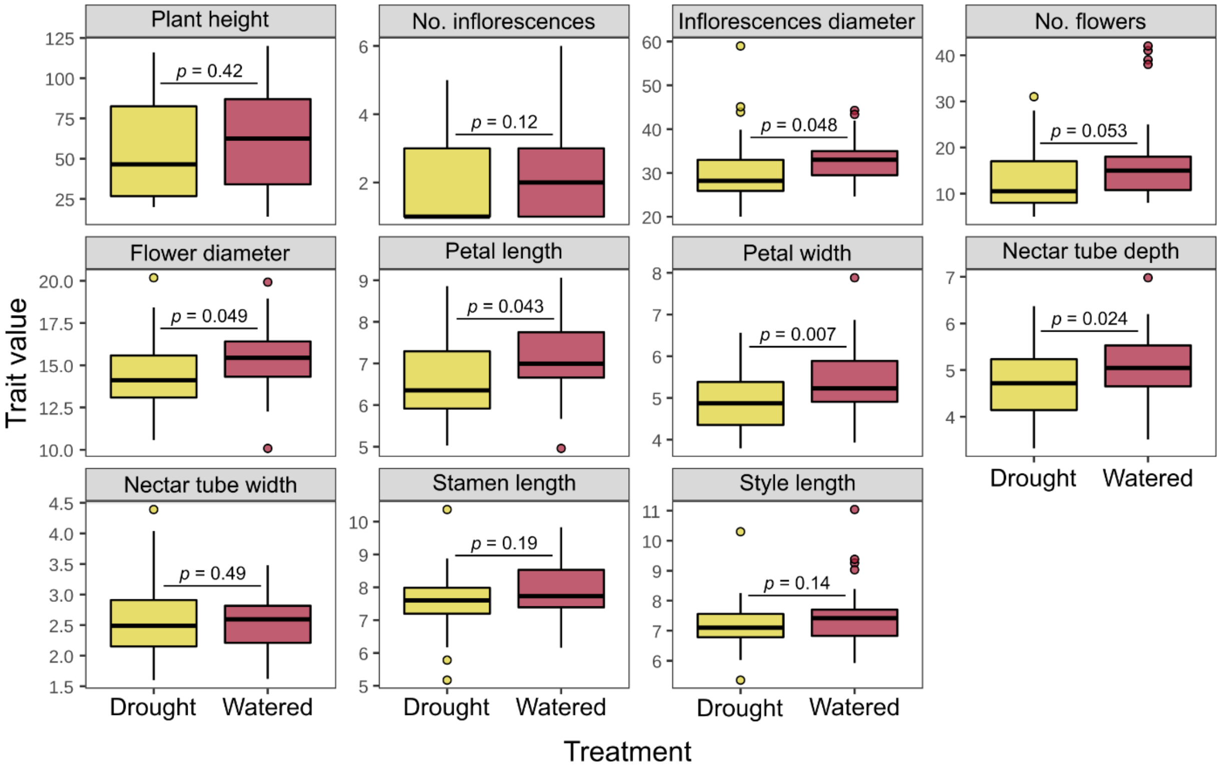

Figure S1.1. Difference between drought and watered treatment in floral trait expressions of *Sinapis arvensis*. Red boxplots = drought treatment, yellow boxplots = watered treatment. Boxplots show median, 25th and 75th percentiles (IQR, boxes), 1.5x IQR (whiskers) and outliers (points). *N* = 36 per treatment. Treatments were compared using generalized linear models with either Gaussian or negative binomial error distribution. All models were calculated in *R* 4.0.2 (R Core Team 2020) using the *glm*()/*glm.nb*() function of the *MASS*-package 7.3-51.6 (Venables and Ripley 2002). Significance was ascertained via Type II Wald chi-square tests implement in the *Anova*() function of the *car* package 3.0-10 (Fox and Weisberg 2019). The fit of each model was assessed using the *DHARMa* package 0.3.2.0 (Hartig 2020) and all assumptions for dispersion, zero-inflation, heteroscedasticity, outliers and deviation from error distribution were met. No. indicates “Total number of”. Trait values for plant height are in centimetres, No. inflorescences and flowers are counts, the rest is in millimetres. Full model results are given in Table S1.1.

Table S1.1. Results of generalized linear models (GLM) testing for differences in floral trait expression of *Sinapis arvensis* between treatments. Estimate shows coefficient and 95% Confidence Intervals; values for models with negative binomial error are back-transformed. $Mean_D \pm SD_D$ = mean ± standard deviation drought treatment. $Mean_W \pm SD_W$ = mean ± standard deviation watered treatment. Trait values for plant height are in centimetres, No. inflorescences and flowers are counts, the rest is in millimetres. Significance was assessed using Analysis of Deviance Table (Type II).

**Trait**	**Estimate**	X_1	***P***	**$Mean_D$ (SD_D)**	**$Mean_W$ ± (SD_W)**
Plant height	1.10 (0.86-1.41)	0.63	0.42	55.86 (30.7)	61.69 (29.75)
Number of inflorescences	1.24 (0.90-1.72)	1.73	0.18	1.83 (1.06)	2.28 (1.39)
Diameter of inflorescences	2.94 (0.03-5.85)	3.92	0.048	29.98 (7.63)	32.92 (4.62)
Number of flowers	1.28 (1.03-1.60)	4.97	0.026	12.97 (6.44)	16.67 (9.25)
Diameter of flowers	0.94 (-0.01-1.89)	3.73	0.053	14.44 (2.16)	15.37 (1.95)
Petal length	0.47 (0.01-0.92)	4.09	0.043	6.69 (1.06)	7.15 (0.90)
Petal width	0.49 (0.13-0.85)	7.24	0.007	4.99 (0.74)	5.40 (0.82)
Nectar tube depth	0.37 (0.05-0.70)	5.10	0.024	4.71 (0.71)	5.08 (0.69)
Nectar tube width	-0.09 (-0.34-0.16)	0.48	0.48	2.62 (0.63)	2.53 (0.45)
Stamen length	0.29 (-0.15-0.73)	1.68	0.19	7.59 (0.93)	7.88 (0.97)
Style length	0.33 (-0.10-0.76)	2.16	0.14	7.14 (0.85)	7.47 (1.03)

Supporting Information 2. Association of interaction with floral expressions for each trait separately.

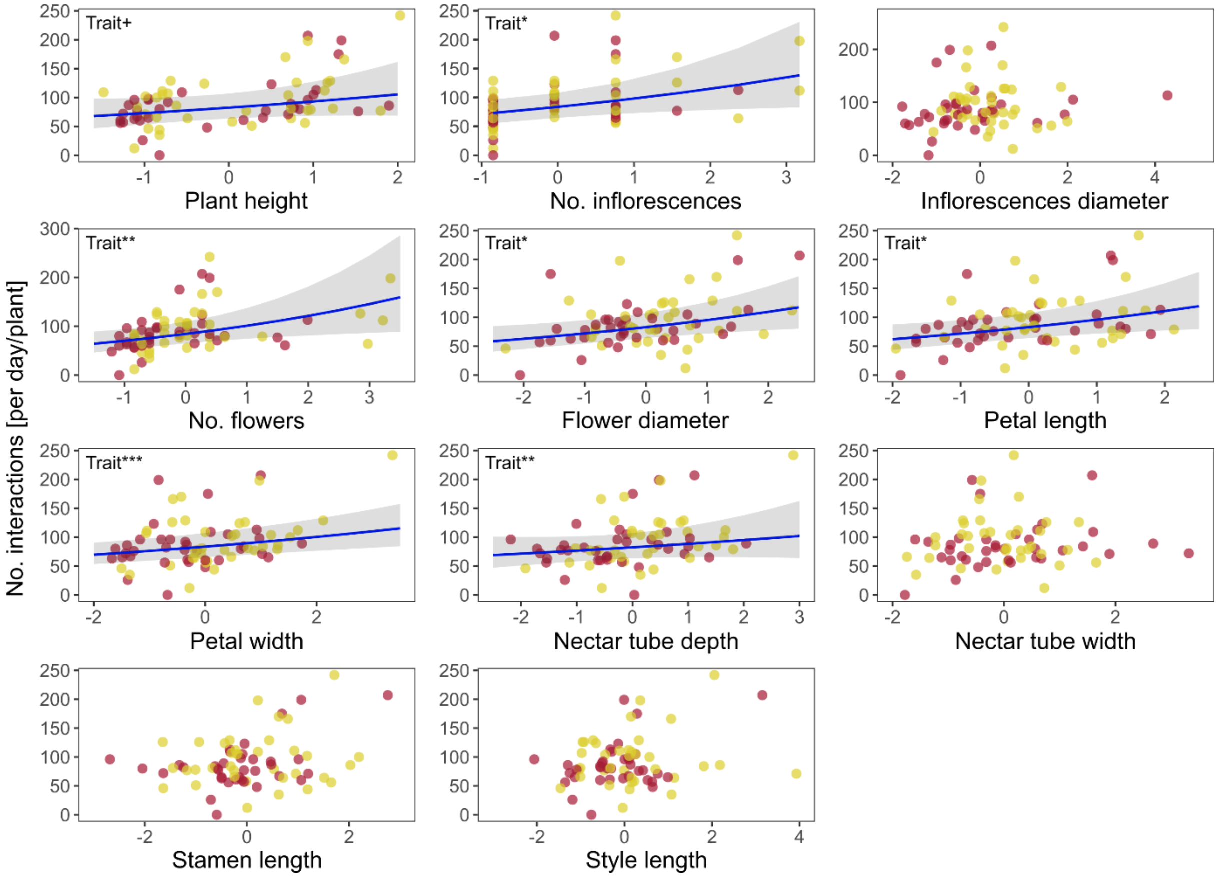

Figure S2.1. Association between floral trait expressions of *Sinapis arvensis* and total number of interactions per day and plant of *Bombus terrestris*. Each red dot represents one plant individual in drought treatment, each yellow dot one in the watered treatment. Blue lines are regression lines derived from (generalized) linear mixed models including trait, treatment and their interaction; grey shades are standard error of the regression lines. The association between number of interactions, floral trait expression and treatment was explored using negative binominal-distributed generalized linear mixed models with treatment, one floral trait and their interaction with treatment as fixed factor and date of sampling and plants within block nested in block as random factors. All models were calculated in R 4.0.2 (R CoreTeam 2020) using the g*lmmTMB*() function of the *glmmTMB*-package 1.0.2.1 (Brooks *et al.* 2017). Significance was ascertained *via* Type II Wald chi-square tests implement in the *Anova*() function of the car package 3.0-10 (Fox and Weisberg 2019). The fit of each model was assessed using the *DHARMa* package 0.3.2.0 (Hartig 2020) and all assumptions for dispersion, zero-inflation, heteroscedasticity, outliers and deviation from error distribution were met. No. indicates "Total number of". All traits values combined were mean-centred and scaled by one standard deviation. Significance levels are given as asterisks: *** $P < 0.001$, ** $P < 0.01$, * $P < 0.05$, + $P < 0.1$ and only significant terms are shown. Full model results show in Table S2.2.

Table S2.2. Results of the generalized linear mixed-models (GLMMs) testing for differences in number of interactions of *Bombus terrestris* with *Sinapis arvensis* for each floral trait separately. Estimate shows coefficient and 95% Confidence Intervals; values are back-transformed.

Trait	Estimate	*z*	*P*	X_1	*P*
Intercept	82.52 (63.59-107.09)	33.20	< 0.001		
Plant height	1.12 (0.96-1.34)	1.43	0.15	3.32	0.07
Treatment	1.07 (0.91-1.25)	0.78	0.44	0.91	0.34
*Treatment*Trait*	1.04 (0.90-1.21)	0.58	0.56	0.34	0.56
Intercept	83.33 (64.49-107.67)	33.82	< 0.001		
No. inflorescences	1.17 (1.02-1.34)	2.27	0.02	6.23	0.01
Treatment	1.05 (0.90-1.23)	0.68	0.49	0.33	0.57
*Treatment*Trait*	0.94 (0.80-1.09)	-0.86	0.39	0.75	0.39
Intercept	82.17 (62.00-108.89)	30.69	< 0.001		
Diameter of inflorescences	1.08 (0.97-1.20)	1.34	0.18	1.62	0.20
Treatment	1.09 (0.92-1.28)	1.00	0.32	0.85	0.36
*Treatment*Trait*	0.96 (0.80-1.16)	-0.42	0.67	0.18	0.67
Intercept	84.14 (64.83-109.20)	33.32	< 0.001		
No. flowers	1.20 (1.04-1.39)	2.45	0.14	6.96	0.008
Treatment	1.04 (0.89-1.22)	0.52	0.60	0.19	0.67
*Treatment*Trait*	0.91 (0.77-1.07)	-1.16	0.25	1.35	0.25
Intercept	82.59 (63.76-106.97)	33.44	< 0.001		
Diameter of flowers	1.15 (1.03-1.28)	2.52	0.01	5.43	0.02
Treatment	1.08 (0.93-1.26)	1.00	0.32	0.68	0.41
*Treatment*Trait*	0.92 (0.78-1.07)	-1.09	0.27	1.19	0.27
Intercept	83.00 (64.06-107.53)	33.44	< 0.001		
Petal length	1.16 (1.03-1.30)	2.37	0.02	5.05	0.02
Treatment	1.09 (0.91-1.25)	0.83	0.40	0.45	0.50
*Treatment*Trait*	0.93 (0.79-1.085)	-0.94	0.35	0.89	0.35
Intercept	83.59 (65.25-107.09)	35.01	< 0.001		
Petal width	1.10 (1.04-1.15)	3.52	< 0.001	34.48	< 0.001
Treatment	1.04 (0.91-1.18)	0.55	0.58	0.61	0.58
*Treatment*Trait*	0.99 (0.93-1.05)	-0.24	0.81	0.06	0.81
Intercept	82.39 (63.41-107.06)	33.02	< 0.001		
Nectar tube depth	1.07 (0.95 -1.21)	1.15	0.25	8.37	0.004
Treatment	1.04 (0.89-1.21)	0.51	0.61	0.30	0.58
*Treatment*Trait*	1.08 (0.94-1.26)	1.10	0.27	1.20	0.27
Intercept	79.37 (61.08-103.12)	32.74	< 0.001		
Nectar tube width	1.10 (0.98-1.24)	1.64	0.10	1.35	0.25
Treatment	1.14 (0.97-1.34)	1.56	0.12	1.75	0.19
*Treatment*Trait*	0.85 (0.67-1.07)	-1.37	0.17	1.88	0.17
Intercept	82.35 (61.28-110.67)	29.25	< 0.001		
Stamen length	1.12 (0.98-1.28)	1.65	0.10	1.81	0.18
Treatment	1.08 (0.93-1.26)	1.03	0.30	1.05	0.31

*Treatment*Trait*	0.92 (0.77-1.09)	-0.96	0.34	0.93	0.34
Intercept	81.84 (61.39-109.09)	30.04	< 0.001		
Style length	1.07 (0.94-1.22)	0.98	0.33	2.07	0.15
Treatment	1.09 (0.93-1.27)	1.04	0.30	1.08	0.30
*Treatment*Trait*	0.99 (0.84-1.18)	-0.06	0.95	0.004	0.95

Each model included one trait, treatment and the interaction between trait and treatment as fixed factors and plants within block nested in block and sampling date as random factors. Significance of fixed effects was assessed with likelihood ratio tests.

References

Brooks ME, Kristensen K, van Benthem KJ, *et al.* 2017. glmmTMB balances speed and flexibility among packages for zero-inflated generalized linear mixed modeling. *R Journal* **9**: 378–400.
Fox J, Weisberg S. **2019**. *An R companion to applied regression*. Sage, Thousand Oaks, CA, USA.
Hartig F. **2020**. DHARMa: Residual diagnostics for hierarchical (multi-level/mixed) regression models. R package version 0.3.2.0. https://CRAN.R-project.org/package=DHARMa
R CoreTeam. **2020**. R: A language and environment for statistical computing.
Venables WN, Ripley BD. **2002**. *Modern Applied Statistics with S.* Springer, New York, NY, USA
